# Root and rhizosphere processes–high time to dig deeper

**DOI:** 10.3389/fpls.2014.00278

**Published:** 2014-06-12

**Authors:** Boris Rewald, Douglas L. Godbold, Omer Falik, Shimon Rachmilevitch

**Affiliations:** ^1^Department of Forest and Soil Sciences, Forest Ecology, University of Natural Resources and Life Sciences Vienna (BOKU)Vienna, Austria; ^2^Mitrani Department of Desert Ecology, Blaustein Institutes for Desert Research, Ben-Gurion University of the NegevMidreshet Ben-Gurion, Israel; ^3^Blaustein Institutes for Desert Research, French Associates Institute for Agriculture and Biotechnology of Drylands, Ben-Gurion University of the NegevMidreshet Ben-Gurion, Israel

**Keywords:** root ecology, botany, rhizosphere, plant growth promoting bacteria, mycorrhizae, earthworms, root physiology, root morphology, soil hydrology, root system architecture, carbon sequestration, root traits, soil science, crop breeding, drought tolerance, heavy metals, lead, aluminum, ectomycorrhizae, root system model, ecophysiology of plants, root dynamics, deep roots, coarse roots

The purpose of the research topic “Ecophysiology of root systems-environment interactions” was to shed light on belowground processes—in an effort to further enhance its understanding, but also to increase the awareness of the research community and funding bodies toward this utmost important part of plants and ecosystems.

Why is it important to increase our understanding and awareness? The new challenge of global climate change is driven by an increase in atmospheric CO_2_ levels, a factor which in itself affects plant and root growth, but is also expected to increase the intensity of climatic and edaphic extremes. In addition, other stresses such as salinization and heavy metal contamination of soils are either increasing or continue to persist world-wide. To secure crop yield and soil quality, and to understand the functioning, and thus the resilience and resistance of pristine ecosystems under changing environmental conditions, an increased understanding of acclimation and adaptation processes is imperative. In the past, plant sciences' research has focused predominantly on parameters above ground—resulting in disproportional less knowledge regarding root systems and the way root system functioning is affected by both internal and external factors. Similarly, soil scientists have often preferred studying bulk soil over the rhizosphere and as a consequence root-driven soil processes are still far less studied. Because it is “better to light a candle than curse the darkness” (Herron et al., 2013), this research topic puts research on root and rhizosphere into the spotlight.

Increased net photosynthesis and decreased shoot nitrogen and water use under elevated CO_2_ can alter source–sink relations of plant organs. In this research topic, Easlon and Bloom (2013) emphasize the important role of root-shoot signaling for plant acclimation to increasing CO_2_ levels. Addressing water availability, Carminati (2013) shows that fast and almost immediate rewetting after soil drought took place in the rhizosphere of distal maize root segments while the rhizosphere of higher root orders possessed slower rewetting. The difference in the speed of rewetting for different root orders, may possibly be an adaptation strategy to drought periods, increasing the water uptake by young root segments and hydraulically disconnecting the older ones. Two comprehensive reviews shed light on the influence and interplay of heavy metals on/with root systems, and influence of root traits on whole plant stress resistance. In one review, Fahr et al. (2013) address the wide range of tolerance mechanism of roots against lead exposure, while in another review Brunner and Sperisen (2013) focus on the current understanding of aluminium exclusion and tolerance mechanisms in woody plants.

The response of roots to abiotic stress can be modified by some root-associated bacteria and fungi. This is expressed as modification of root morphology and whole plant ecophysiology, which often enhances plant growth under stress (Alavi et al., 2013; Vacheron et al., 2013). Increasing knowledge of bacterial nutrition in the rhizosphere will further increase the understanding of the role of certain bacteria as plant-growth promoters (Lopez-Guerrero et al., 2013). Similarly, mycorrhizal symbionts are very important for soil exploration; in this context, Lang et al. (2013) report on spatial structuring of ectomycorrhizal assemblages within beech root systems. While earthworms often improve soil structure and nutrient availability, Arnone and Zaller (2014) report decreasing grass root length densities under increasing earthworm densities, with yet unknown consequences for nutrient foraging.

Besides abiotic stress, root-microbe and root-fauna interactions, plant-plant interactions below ground are common and can influence plant performance considerably. For example, Bolte et al. (2013) show that beech fine roots are facilitated in the presence of spruce roots—possibly by lowering the competitive pressure (for resources) compared to intraspecific competition. While information on the mechanisms of belowground neighbor perception is rare, Schmid et al. (2013) outline that Arabidopsis roots perceive neighboring roots or their associated microorganisms by a mechanism that involves the induction of pathogenesis-related proteins. Their findings reveal that belowground neighbor detection may occur independently of resource depletion, possibly allowing roots to anticipate future competition.

In the past years an increasing awareness has developed that understanding root traits will help to understand plant functioning. Since resources are acquired by the root system, breeding for crops with root traits/phenotypes increasing water and nutrient acquisition should increase yields on infertile soils and under a range of other abiotic stresses such as drought (Comas et al., 2013; White et al., 2013). In doing so, a better understanding of how root and root system traits interact to affect soil resource acquisition is needed (York et al., 2013). In woody plants, our understanding of species- or even variety-specific root trait plasticity under variable environments and the importance of specific traits such as deep rooting (Laclau et al., 2013; Maeght et al., 2013) is even more scarce than in crop plants. A primary reason for this difference in understanding being the challenges caused by more complex, difficult-to-access, perennial root systems. Fortunately, several studies of this research topic shed light on the variability/plasticity of (some) fine root traits of several tree species with ontogeny, and/or under different environmental or management conditions. For example, Noguchi et al. (2013) showed that N-fertilization had a more pronounced effect on *Cryptomeria japonica* root morphology than on root biomass. Studying ectomycorrhizal short roots, Ostonen et al. (2013) found that morphological root traits of late-successional spruce are as plastic as that of pioneer silver birch, and that differences between root traits of the two species was less under more temperate conditions compared to more boreal conditions. The work of Tobner et al. (2013) evidenced that the responses to ontogeny or soil conditions are species but also trait dependent. Hajek et al. (2013) found distinct intraspecific variation in most root traits among seven *Poplar* demes. As highlighted by Fort (2013), their results challenge the existence of well-defined species-specific trait values, but rather highlight the existence of pronounced within-species trait diversity linked to genetic differentiation. While manuscripts in this research topic address a plethora of root traits, increasing efforts are also required to understand root system branching *in situ* and *in silica* (Bodner et al., 2013) and to develop meaningful classification approaches for functional units within root systems. Bodner and colleagues showed that statistical classification methods can integrate knowledge on morphological traits obtained with different methods and at various scales. Currently morphology seems to be the most promising basis for classification approaches due to its wide use. However, the lack of consensus about fine root classification (and a clear nomenclature), and the importance of specific traits constrains the development of a unified framework toward a “root economics spectrum” as was achieved for both leaves (see e.g., Poorter et al., 2014 and references therein) and wood. In addition, more suitable methods are needed allowing advanced root research; here Danjon et al. (2013) describe a modeling approach to estimating root loss during tree root system sampling, and Faget et al. (2013) introduce a combined root fluorescence and planar Optode technique which allows to distinguish between different plant species grown in natural soil and to measure the impact of root (exudates) on the soil environment. Using light-emitting soil microbial “biosensors,” Herron et al. (2013) were able to determine hotspots of microbial growth along the growing root. The growth of these microbial hotspots was supported by high carbon availability.

In addition to articles on basic research which often accentuate the uncertainties in the field of root research, some articles already outline the application of knowledge of root traits in applied plant production systems. The study by Kerbiriou et al. (2013) provides first evidence that “robustness” and head growth rates of lettuce cultivars are related to the size of the root system. Terzaghi et al. (2013) investigated C and N concentrations in *Fagus sylvatica* fine roots in relation to different stand characteristics resulting from conversion of coppiced forests to high forests. The fine-root C:N ratio was higher in coppiced than in converted stands and showed an inverse relationship with fine-root turnover rate, illustrating a significant change of fine-root status under different management practices—likely influencing e.g., the C sequestration potential of stands. Chairungsee et al. (2013) revealed a significant negative correlation between fine root dynamics and production in rubber plantations. Latex harvesting might disturb carbon dynamics in the whole tree, far beyond the trunk; the results emphasize the impact of root systems on the carbon budget and thus yield of tree crops plantations.

When taken as a whole, the 28 contributions to this research topic cover many, although by no means all, aspects of root and rhizosphere research. The number of articles collected within a relatively short period of time, and other recently published special issues addressing root-environmental interactions (e.g., Annals of Botany, 2012, 2013; New Phytologist, 2013), demonstrate that the awareness about root and rhizosphere research and its applicability is rising.

## Conflict of interest statement

The authors declare that the research was conducted in the absence of any commercial or financial relationships that could be construed as a potential conflict of interest.
